# Barriers associated with the public use of sports facilities in China: a qualitative study

**DOI:** 10.1186/s12889-022-14441-w

**Published:** 2022-11-18

**Authors:** Wei Gao, Weisheng Feng, Qianli Xu, Shihui Lu, Keqiang Cao

**Affiliations:** 1 School of Economics and Management, Shanghai University of Sport, Shanghai, 200438 China; 2 Department of Physical Education, Shanghai Dian Ji University, Shanghai, 201306 China

**Keywords:** Grounded theory, In-depth interviews, National fitness, Physical activity

## Abstract

**Background:**

Sports and recreational facilities provide an important community resource where physical activity can be promoted among local residents. However, in China, many sports facilities are not fully open to the public and are often underutilized as essential public services. The purpose of this study was to examine the barriers to public entry from the facility management point of view.

**Methods:**

A qualitative study informed by constructivist grounded theory. Individual interviews were conducted with executive managers, marketing staff, and facilities management personnel (*n* = 15). We took an inductive approach to data synthesis utilizing procedural three-level coding, and used the electronic data management program (NVivo12) to organize the data analysis process.

**Results:**

The 15 participants discussed factors that impacted the sports facilities’ capacity to serve the public. Four key themes emerged from data synthesis: (a) Policy-related restrictions, (b) Management-related factors, (c) Service-related factors, and (d) Supervision factors. Specific barriers affecting facilities’ availability and accessibility involved policies and regulations, administrative pressure, institutional mechanisms, ideas, a professional team, practical ways, content development, schedule setting, spirit building, assessment and supervision, crisis management, and public evaluation.

**Conclusion:**

Findings from this qualitative study provided theoretical ground and empirical support for future research aimed at making sports facilities more accessible to the public in order to maximize physical activity and meet the objectives of the national fitness blueprint in China.

## Introduction

Sports facilities are places that host sports training, sports competition, and physical exercise/physical activity. Specifically, the term refers to enclosed areas such as sports pavilions, stadiums, gymnasiums, swimming pools, basketball courts, football fields, volleyball courts, roller and ice rinks, and other similar places where members of the general public assemble to engage in physical exercise, participate in athletic competition, or witness sporting events [1]. Community- or neighborhood-based sports and recreational facilities provide an important built environmental setting where physical and exercise activities can be promoted for people of all ages [2–4]. In China, most of the sports facilities (95%) are invested by the government or public finance [5]. Since the National Fitness Plan (NFP) became a national strategy in 2014, the central and local governments have asked all kinds of public sports facilities to provide accessibility and availability for the Chinese people [6]. That said, professional sports in China are still relatively underdeveloped and so cannot be the sole support for the operation of large-scale stadiums. Therefore, sports facilities seek to increase utilization and generate revenue by hosting amateur sporting events or performances [7].

Evidence indicates that accessibility of sports facilities is associated with an increased frequency of physical activity and that availability of sports facilities is positively related to changes in physical activity and exercise [3, 4]. In some countries, sports facilities are considered a crucial part of public health or health promotion policies aimed at increasing population levels of physical activity, especially in children, adolescents, and older adults [3, 8, 9]. In China, the National Fitness Plan outlines specific guidelines for making local sports facilities available and accessible to community residents as an essential element of its physical activity strategies, which are designed to promote and improve population health [10, 11]. Considering the large population of many Chinese cities, urban public green space is significantly lacking and open roads are not conducive for safely performing physical activity. Studies [12, 13] have shown that improving accessibility to an exercise facility may lead to an increased level of leisure-time physical activity. Therefore, by making sports facilities or venues available and accessible to the public, it may help promote public health by encouraging local residents to participate in recreational physical activities.

Despite China’s public health policies and initiatives, there are few sports facilities across urban and rural areas that are open to the public for physical activity. A national report revealed that there are currently 3.2 million sports facilities nationwide, showing an increasing trend in the development of sports infrastructure over the past 10 years [10]. However, in China, most sports facilities remain largely underutilized, with only 50% of them fully open to public [11, 14]. Although calls have been made to make more sports facilities accessible to local populations in order to promote physical activity [15–18], there has been little to no progress made toward enacting those calls [16]; in fact, more than 90% of the sports facilities remain rarely used or have not reached their maximum capacity [15]. Therefore, to meet the nation’s physical activity guidelines [10, 11], there is an urgent need to understand barriers and factors that either impede or facilitate the opening of sports-related facilities for public use.

Previous studies have mainly focused on the point of view of the general public and governmental policy requirements [19–22]. Most of them concluded that the government should provide more sports facilities to improve residents’ participation in physical activities as well as reform the role these facilities play in their communities. The public’s perception and sense of gain are the main drivers behind the effort to improve the service quality of public sports facilities. As far as the research goes, there is scant evidence pertaining to the joint associations between public perception and governmental policy requirements. To address this knowledge gap, we conducted a qualitative study aimed at identifying practical barriers and facilitators related to making local and national sports facilities accessible to the public. This study expands on previous research by incorporating perspectives from operators of sports facilities. Specifically, in an exploratory mode, we interviewed a group of facility administers, managers, and marketing directors across seven major metropolitan cities in China, examining their opinions, perspectives, and thoughts on the use of sports facilities in relation to community-wide physical activity promotion.

## Methods

### Study design

This qualitative study utilized an inductive thematic analysis approach involving semi-structured interviews [23]. The research team was comprised of members representing different backgrounds involving social science and physical activity researchers, sports management experts, and physical fitness and activity policy advisors. We derived sensitizing concepts from our own and previous research [16, 18, 24].

Grounded theory generates a theoretical model by observing a study population and developing a comparative analysis of their speech and behavior. In-depth interview is the most commonly used method in classical grounded theory research because open communication between interviewer and interviewee allows participants to fully express their opinions and thoughts. Then, by observing the population, researchers can develop a theory to explain the phenomena of interest [25].

This study formulated relevant questions on the topic, formed an interview outline, adopted semi-structured in-depth interviews, and encouraged participants to share their experiences and thoughts about their work. During this process, the interviewers tried to avoid offering guidance and reminders. After obtaining their consent, some interviewees (*n* = 9) were recorded and transcribed by an audio recorder; others (*n* = 6), who declined to disclose their personal information and affiliations, were not. Other than the audio recording, all 15 interviews were conducted the same. A member of our team wrote the main ideas, thoughts, and opinions of each interviewee on a notepad during the interview. Data from all 15 interviewees were used for subsequent analysis. The interviews were conducted between July and October of 2021. We also sought expert (*n* = 2) opinions from the fields of sports management and physical activity to help improve the validity of our research questions and synthesis of qualitative data.

### Study participants and recruitment

The China Sports Venue Association (CSVA) is a nationally recognized association of sports venues and facilities in the mainland of China. The corresponding author of this study (KC) is the deputy president of the CSVA. Our research team attended the annual meeting of CSVA each year between 2016 and 2019 (the annual meeting has been suspended since 2020 due to COVID-19 precautions) and had communication with many members as well as association leaders and staff. Participants for this study were identified through the CSVA, which oversees more than 100 employees working across six geographic regions in China (Northeast, Northwest, North Central, South Central, Southwest, and Southeast). The CSVA therefore provides us with a general sampling framework and data sources for our study.

We selected our sample based on recommendations from the leaders and staff of the association. Through a combination of convenience sampling and purposive sampling, this study selected 21 participants who are experienced experts (either sports facility general managers, marketing staff, management personnel, or IT personnel). Finally, 15 of the experts agreed to participate in the interviews, while the other 6 declined for personal reasons. The basic demographic information (15 participants) is as follows: Participants included women (*n =* 4) and men (*n =* 11) who work as IT personnel (*n* = 2, no. 9 and 15) or those who work as front-line general managers or marketing staff and facilities management personnel (*n* = 13); participants are middle- or top-level leaders in their respective organizations; participants have been engaged in related industries for 5 to 10 years (*n =* 6), 11 to 19 years (*n =* 3), or for more than 20 years (*n* = 6); the facilities in question are located in eastern, western, mid, and north China, and cities include Shanghai, Hangzhou, Beijing, Wuhan, Chengdu, Chongqing, and Zibo (Table 1).

**Table 1:** Participants’ characteristics

No.	Sex	Location	Role at facility	Working years in this area
1	Female	Eastern China	Regional head	7
2	Female	Eastern China	General manager	6
3	Male	Eastern China	Director	13
4	Male	Eastern China	Director assistant	7
5	Male	Eastern China	Vice-director	23
6	Male	Western China	General manager	21
7	Male	Mid China	Management personnel	22
8	Male	North China	Marketing staff	11
9	Female	South China	IT personnel	9
10	Male	North China	General manager	25
11	Male	Eastern China	General manager	26
12	Female	Mid China	Management personnel	10
13	Male	Mid China	Management personnel	16
14	Male	Western China	General manager	23
15	Male	Eastern China	IT personnel	9

With the help of CSVA leaders and staff, we recruited potential participants by telephone. In consideration of COVID-19 restrictions in their respective cities, as well as the research team’s limited budget, we visited nine of the interviewees’ sports facilities for field investigation and invited them for an individual face-to-face interview. The other six interviews took place over the phone or on WeChat (a very popular social media app in China that can be used as a free telephone). All interviews were scheduled at a time and location that were convenient for participants. All participants were informed that their participation was voluntary, and they were allowed to refuse to answer any question or to end the interview at any time. The research protocol in the study was approved by the Institutional Review Board at the Shanghai University of Sport. The nine interviewees who were invited for an individual face-to-face interview provided consent before the interview began, and the other six provided a scanned version by email or WeChat.

### Data collection

The interviewers created a semi-structured outline platform based on an on-site investigation of sports facilities and a literature review. Individual interviews were conducted by the first author (WG) using the semi-structured interview guide. First, respondents were asked to evaluate the current situation with respect to capacity and accessibility of the sports facilities. Specific questions included: *Can you tell us about the function of the facility (*i.e.*, arena, gymnasium, outdoor field, or stadium)*? *What service features does your facility currently include (*e.g.*, organized sports meets or events)? What do you think about these feature settings (in terms of what?)? How can changes be made to expand service to meet the public’s demands (*e.g.*, parties, exercise, walking)?* A second series of questions prompted interviewees to discuss their beliefs regarding how service could be improved to satisfy the public as well as their views on what barriers and factors would be involved. Specific questions included: *Can you tell us some of the challenges that the facility may face in opening up to the public or barriers that may impede public use? What are the detailed factors, such as law, policies, decisions, funds, personnel systems, or performance rewards? Is the facility being used in the way that it was designed to be used? How does management affect the function of the venue and its public sports service capacity?* Participants were encouraged to tell us about their views regarding potential barriers or other factors that may hinder public use of sports facilities. Interviews were audio-taped with participants’ consent and were transcribed verbatim immediately following the interview; the interview time for each sample was 30 to 90 min. Data collection occurred into two phases: Phase 1 was descriptive (initial sampling), whereas interviews conducted in Phase 2 were intended to provide a deeper understanding and explanation based on what we learned in the earlier interviews (theoretical sampling).

### Data analysis

Data collection and data analysis were conducted in parallel [26]. Analysis began immediately following the transcription of each interview. This key stage included initial, focused, axial, and theoretical coding. The first step involved line-by-line open coding of interviews. The second step was focused coding, which entailed comparison of codes, and data were broken into components of properties and labeled. Through cluster analysis, the categories developed in the first-level coding stage were organically combined, the generic relationship between them was gradually determined, and each main category and corresponding categories were summarized. The third step, axial and theoretical coding, refers to the phase where we identified relationships between the constructed categories [27]. We conducted data analysis using a collaborative team coding process and held meetings to discuss the data analysis every 2 weeks, with 4 meetings in total. We developed a list of coded concepts to help characterize the influential contextual factors. Throughout the process, we used memo writing to inform our analyses. Disagreement over assigning some codes led to further discussion until alternative codes were generated and consensus was reached. During the process, some codes were redefined and others merged. Codes were subsequently aggregated into category and themes. Finally, themes and categories characterizing facilitation of or barriers to implementation were decided on by all authors. We used NVivo12 qualitative research data analysis software (Version 12, QSR International, Melbourne, Australia) to organize and analyze the interview data.

## Results

In order to better serve China’s National Fitness Plan, this study analyzed and explored the barriers that affect public accessibility of sports facilities from the perspective of general managers, marketing staff, facilities management personnel, and IT personnel. In total, 121 codes were discovered, and 12 categories were extracted (Table 2), including policies and regulations, administrative pressure, institutional mechanisms, ideas, a professional team, practical ways, content development, schedule setting, spirit building, assessment and supervision, crisis management, and public evaluation. In the end, we identified four themes (core factors): policy-related restrictions, management-related factors, service-related factors, and supervision factors.

**Table 2:** The four themes, 12 categories, and general descriptions of barriers to the use of sports facilities

Theme	Category	Description
Policy-related restrictions (all interviewees mentioned)	Policies and regulations	Legal protection and legal appeal channels faced by business entities in the operation process, such as legal obligations and legal protection of rights and interests in facing disputes, the continuity and sustainability of the released policies.
	Administrative pressure	Mainly involves interference from administrative aspects, such as the choice of venue design, appearance and functions, mandatory return of business during the contract period, both free and low-cost opening and economic benefits.
	Institutional mechanisms	The systems and mechanisms that affect the functioning of the stadium, such as whether the construction of the stadium takes into account the local economic development conditions, the positioning of the stadium, whether the preliminary design fully considers the subsequent use functions, whether the management and operation team participates in the design, talent incentive mechanisms, and smart stadium construction.
Management-related factors (14 interviewees mentioned)	Ideas	Whether the management team has a clear, long-term, and sustainable plan; whether there is a standardized procedure, relying on the leadership’s personal courage or a mature operation idea.
	Professional team	The industry’s ability to attract talents, the specialization of the team vs. recruit of a professional team, how is the team’s ability?
	Practical ways	Ways to achieve the target function, such as intelligent operation, reasonable separation of powers, and selection of appropriate management methods.
Service-related factors (9 interviewees mentioned)	Content development	The service content provided by the venue, such as realistic project settings, creation and introduction of events, youth training, public service projects, some other special projects.
	Schedule setting	Ways to improve service efficiency, such as optimizing the time arrangement of venues, making full use of venue space, upgrading and improving service capabilities.
	Spirit building	Improve the spiritual connotation of sports to increase the stickiness of the masses to the venue, such as shaping the spirit of sports, immersing public in the feeling of sports activities, creating the venue’s brand.
Supervision factors (13 interviewees mentioned)	Assessment and Supervision	Supervision of venues by superiors, the rationality of assessment content and methods, and the rationality of assessment and evaluation systems.
	Crisis management	The ability to handle crisis events and the impact on the reputation of the venue, such as ticket scalpers, consumer complaints, ability to meet epidemics or public emergencies.
	Public evaluation	People’s satisfaction evaluation of stadiums, overall impression, gap between ideal state and reality.

### Policy-related restrictions

Policy-related restrictions were one of the main barriers mentioned by most facilities operators. Such barriers refer to policies and regulations, administrative pressure, and institutional mechanisms. Regarding policies and regulations, almost all respondents believed that the policies in China were relatively underdeveloped in this area. Sports facilities operators expressed little knowledge about their obligations nor authorities, when facing a series of disputes in regard to the use of facilities to the public (check for accuracy).

“*The government released many policies in the field of sports facilities since 2014; however, most of them are about how to provide accessibility and availability for public use and improve service for mega games but they have less to do with the financial benefit or profit of sports facilities.*” (Participant 3).

“*Our sports center has many inner roads, and some car drivers usually drive through these roads to avoid traffic jams. Sometimes traffic accidents happen, but policemen say these are not public roads and they cannot handle these accidents according to traffic laws. Therefore, we have to act as mediators and even sometimes provide compensation*. *This requires the time and energy of our employees that would otherwise be needed to deliver public services*.” (Participant 4).

Furthermore, some of the participants felt inconsistency among some of currently issued facility use policies.

“*In 2016, the government released a policy that states sports facilities have to remove commercial businesses such as restaurants, shops, bars, conference rooms, and management offices in order to allocate more space for sports and exercise. However, recent policies encouraged us to develop sports complexes that include multiple services like those business that we have removed*.” (Participant 11).

As most of the large-scale sports facilities were built by the government. It therefore determines the appearance and style of sports facilities, especially main stadiums and arenas, even though most government officials have little expertise in this field. In many cases, the facilities were intended to host specific large-scale sporting events. Since the primary goal was to hold a particular event, questions about how to serve the general public and encourage physical activity participation afterward were considered secondarily. In terms of administrative pressure, 12 of the respondents believed that the administrative intervention of facilities managers would have a positive effect on the facilities’ service to the public.

“*The city mayor or officers sometimes take the featured arenas as their political achievements*. *Therefore, the design and construction of large public sports facilities often overemphasize its appearance and managers’ voices are weighted less important. At the end, these facilities become city’s landmark buildings at the expense of fitness function or health promotion.*” (Participant 6).

This issue is also reflected in the construction of large-scale facilities in some small- or mid-sized cities that host or co-host the National Games, Provincial Games, and other mega events with administrative goals.

“*Our sports center was constructed to co-host the National Games, and this is an international standard stadium, but our city has no professional clubs. The facilities could only host mass sporting games or events; therefore, they are operating at a loss*.” (Participant 10).

The dislocation between demand and supply may lead to a huge secondary investment in fitness-oriented transformation. However, it is rare that policy is made with respect to the operation mechanism in a way that leads to improving transparency.

“*We do not have full autonomy or initiative in management or business activities, and due to the unstable policies and strict financial disciplines, we need to be very careful with the capital investment for the upgrading of sports facilities*.” (Participant 6).

“*We need to get approval from the government to host some events, like social events, concerts, exhibitions. And mostly, because of the policy limit, our staff has no performance incentives for these events*.” (Participant 10).

Because of the special political and social system of China, the policy-related experiences of Western countries in this field are inapplicable, and participants’ demands and managers’ voices have limited influence on the government-oriented sports facilities.

### Management-related factors

The management-related factors are mainly reflected in three aspects: management authority, management team building, and management mode selection. In terms of management authority, 14 of the respondents said that these public facilities are owned by the government and are specifically devoted to competition or planned events.

“*In past years, we have had no management authority to make decisions whether facilities should be open to the public, let alone when and how. And even under the new policies that the facilities have to open to the public, most of the management programs need to strictly follow the instructions of the government and offer little autonomy*.” (Participant 4).

“*Our main stadium is assigned to the local city team for training, and this occupied most of the opening hours, and all the facilities and services are free. Therefore, we have very limited open hours to acquire revenue.*” (Participant 14).

Almost all respondents mentioned that a professional team (staff) is a basic requirement for the effective management of a sports facility. However, some interviewees pointed out that the marginal positions and low salaries of sports facilities staff make it difficult to attract professional management personnel or high-talent people to this profession.

“*In the building of a professional team, we cannot motivate the team’s enthusiasm and creativity with performance incentives, especially when it comes to additional expansionary events. Competent managers or workers are often poached by private venues or facilities.*” (Participant 6).

“*To be honest, some of the management personnel is derived through nepotism, and they have little expertise in this field. And regarding the new recruits, the candidates sometimes need to pass a strict exam that is organized by the government, but most of the exams have no relationship to expertise in the field of sports facilities management*.” (Participant 5).

In terms of management mode, 94% of sports facilities in China are owned by the government, and 80% of them are operated by a team within the government. Thirteen of the interviewees mentioned that management mode is a key point in the effective management of these facilities.

“*The efficiency of the team is low. They usually obey the guidelines of the government very cautiously*. *But China is a large country, and every province and city has its own features. The management team needs to have the autonomy to lay down their handbook to meet the guidelines, I think.*” (Participant 8).

“*Some researchers in this field introduced the Build-Operate-Transfer (BOT), Build-Transfer (BT), Transfer-Operate-Transfer (TOT), Build-Transfer-Own (BTO), or Renovate-Operate-Transfer (ROT) modes to us, and I had an on-site investigation overseas; however, the acclimatization is difficult, I think*.” (Participant 4).

### Service-related factors

The service factors that affect facilities’ accessibility mainly involve demand-side content, such as recreational sporting events, public services, youth training, and reasonable time settings that the facilities can provide. Their direction and purpose, including program development and time setting, are culturally specific.

“*We have begun to work with some recreational sports clubs or associations that have expertise in related fields to develop some exercise programs for people of all ages*.” (Participant 1).

“*How to attract teenagers and children to come into facilities and enjoy exercise is an important direction for sports facilities, and it is a good way to inspire their interest in sports participation. Basketball, football, tennis, badminton, as well as fencing, martial arts, and taekwondo are all very popular in China.*” (Participant 2).

Even with the accelerated aging of the population of China, the sports participation potential of the elderly has not yet been developed. About 8 of the respondents mentioned that services for the elderly are also one of the focuses of their sports facilities.

“*The older adults’ hobbies and time preferences, organizational forms, and consumption capacity affect the activity forms of the facilities. The time arrangement of facilities is a key factor affecting the facilities’ accessibility to the public, especially on weekends and holidays, for older adults and young athletes. How to make a suitable arrangement? That is the question*.” (Participant 13).

Half of the respondents mentioned that the lack of cultural specificity in sports facilities affects public participation. One of the most important functions of the facilities is to serve nearby communities; therefore, the facilities should not only be used as a space for exercise, but also as a hub for the daily life of residents and a place to encourage sports culture within the community.

“*Chinese sports facilities are still relatively lacking in spiritual shaping, sports culture development, immersive sports environment creation, cultural and creative products. We need to do a lot of work to increase people’s sports participation stickiness and engagement*.” (Participant 9).

### Supervision factors

Supervision factors include work supervision for facilities, performance appraisals, year-end assessments, attendees’ complaints, safety incidents, reports, scalpers, and the public’s satisfaction evaluation. In China, supervision mainly comes from the government, not from the market or professional associations. Although these factors do not directly affect facilities’ accessibility to the public, they can shift or change the work focus of the stadium management personnel, causing them to pay close attention to governmental public relations, thereby indirectly affecting the work of the facilities. These factors primarily include three aspects: assessment and supervision, PR crisis, and public evaluation.

In terms of assessment and supervision, 13 of the interviewees said they believe supervision, assessment, and evaluation of the work on national fitness largely determine the directions and measures implemented by facilities.

“*Assessment and supervision pertain to the free and low-cost opening hours, economic benefits, and the use of funding. They directly affect the facilities managers’ aims and scope. And the frequency of evaluation and supervision methods also greatly squeezed the working time of general managers and personnel, which in turn affects their input in professional business*.” (Participant 3).

With regard to PR crises, half of the facilities managers think that a crisis of public relations limits their work input. This situation is especially common in large- and medium-scale public sports facilities.

“*The elderly usually complains about the fees charged, and younger parents complain about the facilities’ poor conditions; and some private facilities managers may complain that the public facilities are low-priced and [so are] vicious competition. [There has been] public dissatisfaction caused by the requisition or restricted access to facilities due to the COVID-19 pandemic since 2020, and the venues are requisitioned for vaccination or other tasks*.” (Participant 7).

“*Crisis management often occupies much of the time of managers and personnel, and such problems are extremely difficult to solve in a short period, resulting in continuous troubles, making it difficult to concentrate on the business*.” (Participant 3).

As far as public evaluation goes, although there are differences from other fields, sports facilities are often compared with public service departments such as hospitals, libraries, museums, and restaurants.

“*Although the sports facilities differ from other public service departments, many people compare us with them—e.g., information convenience, detailed services, service capabilities and attitudes, public satisfaction, and other aspects. It results in low evaluation, and we need to face the public pressure*.” (Participant 2).

## Discussion

The China National Fitness Plan and “Healthy China 2030” are seeking to create more opportunities for physical activity among the public. The government can improve living conditions for those who live in residential areas by increasing accessibility to public sports facilities [22]. Opening more facilities and providing high-level service to the public is not only the national request, it is also prompted by the need to reduce medical costs [28], especially for children and older adults [29, 30]. However, there is a gap between demand and supply with respect to how the sports facilities provide availability, accessibility, and improved service to the public. Unfortunately, the voices of sports facilities managers and personnel are rarely part of the conversation.

This study examined various barriers that may limit the capacity for sports facilities to provide greater opportunity for physical activity participation among residents of the Chinese public. Through a qualitative analysis, we found barriers mainly in the areas of policy-related restrictions, management, services, and supervision. Findings from our study highlighted the public health challenges that exist for sports facilities as well as for the national fitness system. The “National Fitness Law,” released by the National People’s Congress of China, is a timely guide for modernizing the national fitness governance capacity, which defines the rights and obligations of sports facilities and lays a solid foundation for the law [31].

The government influences public utilization of sports facilities through management, governance, and budgetary policies, which, in turn, guide the implementation strategies chosen by facility management [32]. Between 2014 and 2020, the Chinese government released several policies pertaining to the use of sports facilities for the public. However, few operational details have been provided by these policies to guide implementation in practice. With regard to administrative interferences, this study suggests the government ought to reduce its intervention into the way these facilities do business. To reduce the occurrence of unexpected events or activities at major sports facilities, the government should set a detailed schedule in advance for mega events like the Olympic Games, National Games, or Provincial Games.

Based on reforms in areas such as finance, development potential, and promotion of social recognition, sports facilities either need to enhance the abilities of their management and operation teams through team building, or they need to recruit professional teams. In particular, teams need to form standardized operation modes in line with the economic and living conditions of local cities and communities. Sports facility operators need to have the authority to establish an organizational culture of environmental and social responsibility, internal stakeholder pressure, financial cost–benefit, competitiveness, and ethical motives [33].

Sports facilities could fully cooperate with schools to attract intra-school and inter-school sporting events to public facilities on school days. They could also collaborate with organizations such as the Senior Sports Association or the Legislative Council to explore methods and modes of serving the physical activities of elderly fitness enthusiasts. Finally, in terms of evaluation, the government needs to establish a scientific mechanism to provide feedback on the results.

There are previous studies from China examining the associations between availability of sports facilities and levels of physical activity; however, these studies have mainly focused on the point of view of the general public and governmental policy requirements [19–21]. They have explored the public’s degree of mastery and satisfaction with public sports resources [19], the role of local governments in stadium reform [20], and the means for promoting improvements in service quality at large public stadiums [21]. A strength of our study was that it included a range of interdisciplinary perspectives represented by sports facilities managers, who have rich operational experience in this field and work daily to improve the management quality of sports facilities. We established clear processes for team engagement throughout all phases of the study. These were essential as we progressed through the data analysis phase using grounded theory. We suggest that future researchers undertaking such an endeavor establish and document the processes for team engagement, collaborative approaches to data analysis, and preparation of knowledge translation materials early in the study design. A limitation of this study is that its focus was on a single model of general factors. In China, which is a huge country, cities and provinces have differing socioeconomic statuses, demographics, exercise hobbies, habits, and so on; therefore, our findings may not apply to all cities’ sports facilities. Additionally, the method of recruitment of participants in this study generates a potential self-selection bias of those with a particular interest in this area. The sample of 15 interviewees is also rather limited in number, but because of budgetary and travel limitations, we could not interview all participants face to face.

## Conclusion

In a qualitative analysis of perspectives and opinions among sports facility managers and operators, we identified four major barriers that may have restricted the effort to make sports facilities fully accessible to the public in China. We concluded that sustained and implementable policies are needed to make local sports facilities available and fully accessible to the public and to achieve the public health goals set by the National Fitness Plan. Operators of facilities should work on long-term planning, building a professional team, and finding other practical ways to promote services to their communities. Finally, it may be necessary to form a tripartite evaluation system, wherein the government, facilities operators, and the general public cooperate in the assessment and supervision of sports facilities reform.

## Data Availability

The datasets generated and/or analyzed during the current study are not publicly available due participants’ request but are available from the first author on reasonable request.
